# WALKING AND TALKING ABOUT WHAT USED TO BE: THE SHARP NEIGHBORHOOD WALKING PROGRAM FOR OLDER AFRICAN AMERICANS

**DOI:** 10.1093/geroni/igz038.1907

**Published:** 2019-11-08

**Authors:** Juell Towns, Patrice Fuller, Edline Francois, Raina L Croff, Jeffrey Kaye

**Affiliations:** 1 Portland State University, Portland, Oregon, United States; 2 Oregon Health & Science University, Portland, Oregon, United States

## Abstract

The Sharing History through Active Reminiscence and Photo-Imagery (SHARP) study aims to preserve African American cognitive health through neighborhood walking and social engagement in a way that celebrates Black culture. For 6 months, African Americans aged 55+ (2016 n=19; 2017 n=21) grouped in triads walked 1-mile routes accessible via the SHARP application. Routes included historical image prompts about Portland, Oregon’s historically Black neighborhoods. Participant focus groups at months 1, 3, and 6 drove program development and refinements, and provided valuable insight into the program’s meaning for participants. Discussions were thematically coded. Emergent themes included “suggested improvements,” “technology,” “mental health impact,” “cultural incongruence,” and “cultural significance.” Participants suggested improvements to the application’s navigational aspects and expressed willingness to engage technology despite initial apprehension. The triadic structure and place-based memory prompts aided reminiscence, allowing participants to make meaningful links between their own life experiences and their walking partners’. Neighborhood walking brought to the surface participant concerns about a lack of understanding between African American generations, and between long-time residents and whiter, wealthier demographics moving in. Some participants found it emotionally taxing to walk in the now gentrified historically Black neighborhoods, but still saw the program overall as useful, interesting, and necessary--to their physical and cognitive health, to their mental health as they processed neighborhood changes and community loss, and as an important contribution to preserving community history. Addressing individual health alongside pressing issues affecting older African Americans’ sense of well-being and community may make cognitive health programs more meaningful and applicable.

